# Cutaneous Rosai – Dorfman disease in a patient with late syphilis and cervical cancer – case report and a review of literature

**DOI:** 10.1186/s12895-020-00115-w

**Published:** 2020-12-07

**Authors:** Angelika Bielach – Bazyluk, Agnieszka B. Serwin, Agata Pilaszewicz – Puza, Iwona Flisiak

**Affiliations:** 1 Department of Dermatology and Venereology, Medical University of Bialystok, Białystok, Poland; 2 Department of Medical Pathomorphology, Medical University of Bialystok, Białystok, Poland

**Keywords:** histiocytosis, syphilis, Rosai-Dorfman disease, emperipolesis

## Abstract

**Background:**

Cutaneous Rosai – Dorfman disease (CRDD) is extremely rare variant of idiopathic histiocytic proliferative disorder, which may manifest as a non-specific macules, papules, plaques or nodules ranging in size and colour from yellow – red to red -brown.

**Case presentation:**

A 52-year-old female presented with three gradually enlarging, reddish - brown nodules on the right upper extremity lasting six months. The patients denied fever, weight loss, malaise. Clinical examination and imaging tests showed no sign of lymphadenopathy. A biopsy specimen of a nodule showed a dense dermal polymorphic infiltrate with numerous histiocytes exhibiting emperipolesis phenomenon. Immunohistochemical staining of the histiocytes showed S-100 protein (+), CD68(+), but CD1a (-). Aforementioned findings were consistent with CRDD characteristics. Additionally, a routine serological screening and confirmatory serological tests for syphilis were positive. Syphilis of unknown duration was diagnosed. The IgG antibodies titre against Chlamydia trachomatis was elevated. An isolated sensory impairment over the right trigeminal nerve was found on neurological consultation. Comprehensive gynaecological assessment was carried out because of patient’s complaints of bleeding after sexual intercourse and led to diagnosis of cervical cancer. The initial therapy with methotrexate was discontinued after three months due to neutropenia. Further therapy with dapson was ineffective, therefore complete surgical excision was recommended.

**Conclusions:**

CRDD is a rare, benign condition especially difficult to diagnose due to lack of general symptoms and lymphadenopathy. Histopathologic examination with immunohistochemical staining, exhibiting characteristic and reproducible findings play a key role in establishing an accurate diagnosis. In the presented case activated histiocytes demonstrated in a lesional skin might be a response to immune dysregulation related to chronic, untreated sexually transmitted infections and cancer.

## Background

Rosai – Dorfman disease (RDD) is a benign histiocytic proliferative disorder of not fully elucidated aetiology, usually presenting with massive, bilateral, painless cervical lymph nodes enlargement and general symptoms [1]. Internal organ involvement is presented in 43% of cases, while skin is affected only in 10% patients with systemic form [2]. Over the last decade RDD limited to the skin (cutaneous RDD – CRDD) has been increasingly reported. Although histopathological picture and immunohistochemical staining are distinctive and reproducible feature of the disease and allows to establish accurate diagnosis in any site of involvement, the aetiology still remains debatable. Association with HHV-6, HIV, HSV, VZV, Parvovirus B19 and EBV infection was reported, but it seems to be a non-specific reactive response as viral genomes are frequently detected in disorders of lymphoid tissue [1, 3]. Several reports described co-existence of autoimmune diseases and neoplasms with RDD [1, 3]. To the best of our knowledge it is the first case of CRDD reported internationally from Poland and co-existing with sexually transmitted infections and cervical cancer.

## Case presentation

**Fig. 1:**
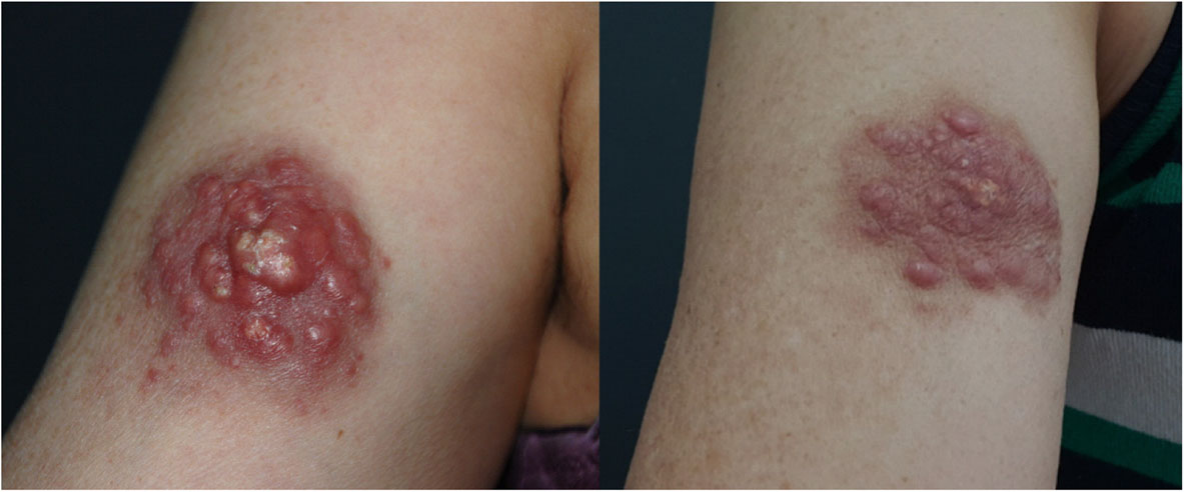
Clinical manifestation on admission and after 3-months therapy with methotrexate 15 mg/week

**Fig. 2:**
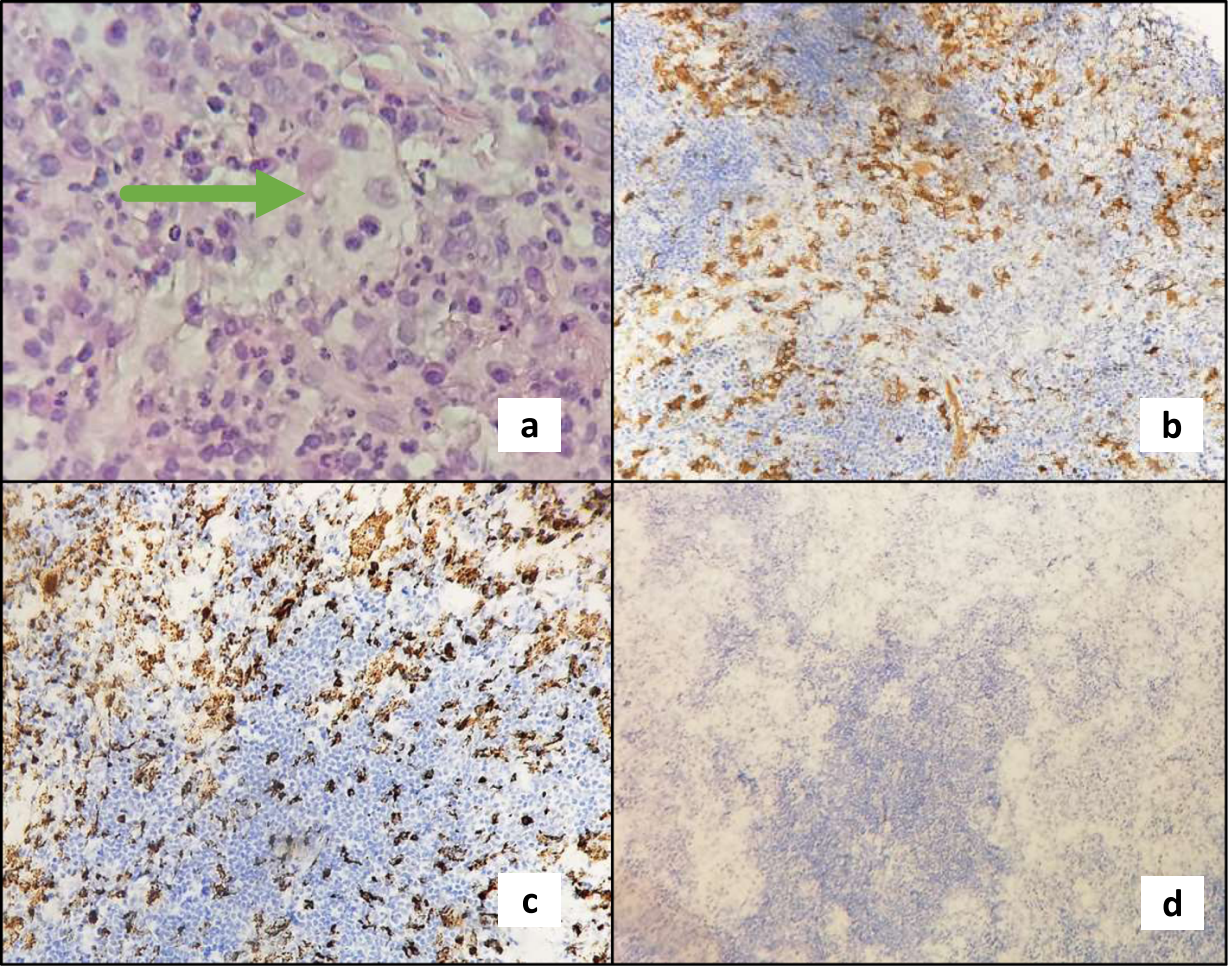
**a** -Polymorphic inflammatory infiltrate with a large histiocyte with bright, abundant cytoplasm exhibiting empreipolesis phenomenon (green arrow) (H&E original magnification 600x); **b** - Positive reaction with S100 protein in histocytic cells, (original magnification 200x); **c** - Positive CD68 reaction in histiocytic cells, (Original magnification 200x); **d** - Negative reaction with CD1a, (Original magnification 100x)

A 52-year-old Caucasian female was admitted to the Department of Dermatology and Venereology because of a single, painless, gradually enlarging, reddish - brown nodule on the right arm accompanied by two small indurated plaques lasting six months (Fig. 1a). On dermatological examination a firm, dome-shaped nodule reaching 7 cm in diameter located on the lateral area of the right arm was found. The surface of the lesion was micronodular with diaphanous yellowish content inside. Additionally, two solitary, round erythematous, indurated plaques 1,5 cm in diameter were found on the right forearm and posterior area of the arm. The patients denied fever, weight loss, malaise. A biopsy specimen of the skin nodule showed a dense dermal polymorphic infiltrate composed of lymphocytes, plasma cells, neutrophils, eosinophils and numerous histiocytes with abundant pale pink cytoplasm exhibiting emperipolesis phenomenon (Fig. 2a). Immunohistochemical staining of the histiocytes showed expression of S-100 protein (+), CD68(+), but CD1a (-) (Fig. 2b-d). Clinical examination, chest X-ray, abdominal ultrasound showed no sign of enlarged lymph nodes. On the basis of the aforementioned findings a diagnosis of cutaneous Rosai – Dorfman disease was made.

Additionally, a routine serological screening test for syphilis gave positive result and was confirmed by positive TPHA (1:20,480) and weakly positive VDRL (1/4). The patient claimed to be never diagnosed nor treated for syphilis previously. IgG anti – *Chlamydia (C.) trachomatis* antibodies were also elevated, HIV test was negative. Other routine laboratory test results were within normal range. No abnormalities on cardiological consultation were found. A sensory impairment in the skin enervated by a maxillary branch of trigeminal nerve was demonstrated on neurological examination and lumbar puncture was recommended. Because of patient’s refusal to the procedure, magnetic resonance of the brain was performed and showed uneven outline of the left cerebellopontine angle; contrast agent injection excluded focal lesions.

The patient complained also of bleeding after intercourse lasting three weeks. Her last Pap smear was performed 20 years ago. The gynaecological consultation revealed nascent uterine myoma, while cervical cytology revealed the presence of low-grade squamous intraepithelial lesion and atypical glandular cells. Further diagnostic and therapeutic procedures included endometrial abrasion and cervical biopsy. Cervical cancer G3, stage IB1 according to FIGO was confirmed by histopathological examination and postoperative assessment. Patient underwent Wertheim’s hysterectomy.

The treatment with doxycycline 100 mg twice daily for 28 days was started, then low-dose methotrexate (MTX, 15 mg/week) was introduced and resulted in partial improvement (Fig. 1b). The treatment with MTX was ceased after three months due to neutropenia possibly in the course of the methotrexate treatment and postoperative radiotherapy. Further regimen included dapson 100 mg daily, but skin lesions showed no improvement after three consecutive months, therefore complete surgical excision was recommended. The procedure have not be carried out, so far, due to coronavirus disease (COVID-19) outbreak. Patient’s husband had also positive syphilis serology and was treated with doxycycline. Both completed appropriate after-treatment follow-up.

## Discussion and conclusions

Cutaneous involvement associated with Rosai - Dorfman disease was described for the first time in 1978 [4]. It is estimated that the disease is limited to the skin only in three percent of cases [2, 5]. Some authors distinguish purely CRDD and CRDD with systemic involvement [1]. On the ground of the clinical findings, histopathological picture and other laboratory investigations we could diagnose in our patient purely CRDD. CRDD may manifest as non-specific macules, papules, plaques or nodules ranging in size and colour from yellow – red to red – brown [1]. Lesions imitating acne, vasculitis or panniculitis was also reported [6–8]. Purely CRDD affects predominantly middle-aged Asian women, followed by Caucasians ones [1]. Similarly to data from comprehensive assessment of over 200 CRDD cases, also in our patient extremities were the location of lesions [1].

Doubtlessly, numerous uncharacteristic cutaneous presentation and lack of specific laboratory findings makes the diagnosis of CRDD challenging. Other histiocytosis, sarcoidosis, granulomatous infectious diseases, lymphomas and soft tissue tumours are most commonly considered in clinical differential diagnosis. Histopathological and immunohistochemical examinations, however, present reproducible features regardless site of involvement are crucial for making correct diagnosis[1]. On histology, the epidermis is unaffected, but the dermis and subcutaneous tissue is densely infiltrated by large histiocytes with abundant pale cytoplasm mono- or multinuclear nuclei and considerable nucleoli, accompanied by scattered lymphocytes, neutrophils and plasma cells [1–3]. Macroscopic yellowish appearance and nodular surface of the lesions derives from clusters of foamy cells and increased stromal reaction, respectively [1]. The most characteristic, but not pathognomonic histologic finding is emperipolesis phenomenon relying on the presence of intact lymphocytes, plasma cells or erythrocytes inside histiocytes. However, it is less frequently seen in extra-nodal sites [1–3]. Moreover, the engulfment of living cells can be seen in other neoplastic (haematolymphoid disorders), inflammatory (autoimmune hepatitis) and genetic disorders (H syndrome) [5, 9]. Occasionally, as it was demonstrated in our patient, the presence of oedematous endothelial cells may mislead to syphilis diagnosis [10]. In case of CRDD suspicion immunohistochemical staining should always be carried out. The combination of expression of S-100(+), CD1a(-), CD68(+), CD163(+) is pathognomonic for RDD [1–3]. The absence of CD1a and Birbeck granules allow to distinguish between RDD and potentially lethal Langerhans histiocytosis [1].

At present, the theory of immune system dysregulation with altered cytokine expression secondary to infectious and neoplastic processes is postulated in CRDD aetiology [1]. It has been hypothesized that histiocytic infiltrate is a consequence of excessive macrophage colony stimulating factor (M-CSF) in the presence of infection [1]. As the patient refused parenteral treatment, we prescribed doxycycline to cover both *Treponema pallidum* and possible *C. trachomatis* infections. Doxycycline is also a therapeutic option in late syphilis according to the latest ‘2020 European guideline’ [11]. Because of unknown duration of the disease and inability to definitely rule out neurosyphilis a 28 – day treatment was administered [12, 13]. Given the fact that Human Papilloma Virus (HPV) is considered as necessary agent in the development of cervical cancer [14], it can be assumed that HPV infection also co-existed, however molecular testing was not performed. Worryingly, the neglecting of gynaecological examination and Pap smear screening on regular basis can still be seen among Polish females [15].

Due to the rarity of the disease and unknown aetiology only empirical therapeutic modalities based on case reports are available. CRDD has an indolent course and occasionally heal spontaneously, which may support the use of expectant attitude in management for this condition [3, 5]. However, in patients with solitary lesions surgical removal is preferred, with 59% of complete remissions [1]. Other reported treatment approach include: dapson, thalidomide, retinoids, methotrexate, steroids, radiotherapy, cryosurgery and corticosteroids achieving the lowest response rate [1–3, 5–7]. In the presented case the treatment with MTX resulted in partial improvement after three months. On the basis of previously reported cases we can suspect that prolonged drug administration may be essential for complete remission [16]. Dapson was ineffective despite dense neutrophilic infiltrate in the biopsy specimen.

We would like to highlight the importance of consideration of rare entities in the differential diagnosis of non-specific skin lesions, especially since the proper one could be made only on the basis of advanced, not widely used techniques such as immunohistochemistry. On the basis of examination performed we can assume that we report a case of purely CRDD in a woman with complex medical history involving several sexually transmitted infectious diseases, malignancy and, ultimately, unexplained neurologic symptoms. Initially, our report seemed to support hypothesis that CRDD may be a response to the immune dysregulation, in this particular case, related to chronic co-existing sexually transmitted infections and neoplastic process. It could be expected that skin lesions will resolve after treating the infections and hysterectomy. However, the patient fail to respond to the treatment applied, rendering the hypothesis questionable. CRDD with limited lesions may benefit more from surgical intervention than pharmacotherapy.

## Data Availability

Data sharing is not applicable to this article as no datasets were generated or analyzed during the current study.

## Abbreviations

CRDD: cutaneous Rosai–Dorfman disease
RDD: Rosai–Dorfman disease

## References

[1] Ahmed A, Crowson N, Margo CM. A comprehensive assessment of cutaneous Rosai-Dorfman disease. Ann Diagn Pathol. 2019 Jun;40:166–73. 10.1016/j.anndiagpath.2019.02.004 31108464

[2] Farooq U, Chacon AH, Vincek V, Elgart GW. Purely cutaneous rosai-dorfman disease with immunohistochemistry. Indian J Dermatol. 2013 Nov;58(6):447–50. 10.4103/0019-5154.119953 PMC3827516 24249896

[3] Frater JL, Maddox JS, Obadiah JM, Hurley MY (2006). Cutaneous Rosai-Dorfman disease: comprehensive review of cases reported in the medical literature since 1990 and presentation of an illustrative case. J Cutan Med Surg.

[4] Tanaka N, Asao T. Sinus histiocytosis with massive lymphadenopathy (Rosai and Dorfman) and significant skin involvement. Acta Pathol Jpn. 1978 Jan;28(1):175–84. 10.1111/j.1440-1827.1978.tb01258.x 636879

[5] Landim FM, Rios Hde O, Costa CO, Feitosa RG, Rocha Filho FD, Costa AA. Cutaneous Rosai-Dorfman disease. An Bras Dermatol. 2009 Jul;84(3):275–8. 10.1590/s0365-05962009000300010 19668942

[6] Zhang Y, Chen H, Image G. Generalized cutaneous Rosai–Dorfman disease presenting as acneiform lesions. Br J Dermatol, 180: e36-e36. doi: 10.1111/bjd.17260. 10.1111/bjd.17260 30714099

[7] Puppin D, Chavaz P, Harms M (1992). Histiocytic Lymphophagocytic Panniculitis (Rosai-Dorfman Disease): A Case Report. Dermatology.

[8] Stefanato CM, Ellerin PS, Bhawan J. Cutaneous sinus histiocytosis (Rosai-Dorfman disease) presenting clinically as vasculitis. J Am Acad Dermatol. 2002 May;46(5):775–8. 10.1067/mjd.2002.119565 12004323

[9] Molho-Pessach V, Agha Z, Aamar S, Glaser B, Doviner V, Hiller N (2008). The H syndrome: A genodermatosis characterized by indurated, hyperpigmented, and hypertrichotic skin with systemic manifestations. J Am Acad Dermatol.

[10] Plaza JA, Prieto VG. Infectious diseases of the skin In: Plaza JA, Prieto VG, Suster S. Inflammatory skin disorders. Demos Medical;2012. p. 191–234.

[11] Janier M, Unemo M, Dupin N, Tiplica G, Potočnik M, Patel R. 2020 European guideline on the management of syphilis. J Eur Acad Dermatol Venereol. 2020 Oct 22. doi:10.1111/jdv.16946. Epub ahead of print. 10.1111/jdv.16946 33094521

[12] Dai T, Qu R, Liu J, Zhou P, Wang Q (2016). Efficacy of Doxycycline in the Treatment of Syphilis. Antimicrob Agents Chemother..

[13] Kang-Birken SL, Castel U, Prichard JG (2010). Oral doxycycline for treatment of neurosyphilis in two patients infected with human immunodeficiency virus. Pharmacotherapy..

[14] Goodman A (2015). HPV testing as a screen for cervical cancer. BMJ.

[15] Skorzynska H, Krawczyk-Suszek M, Kulik TB, Pacian A, Stefanowicz A, Skowronek A (2017). Attitudes of women after the age of 50 towards preventive screening. Ann Agric Environ Med.

[16] Sun NZ, Galvin J, Cooper KD (2014). Cutaneous Rosai-Dorfman Disease Successfully Treated With Low-Dose Methotrexate. JAMA Dermatol.

